# Self-Help and Co-Operation in Institutional Economy

**Published:** 1912-12-07

**Authors:** 


### SELF-HELP AND CO-OPERATION IN INSTITUTIONAL ECONOMY.

### I.—The Question of Initial Expenses.

In former issues of The Hospital we have anticipated a future policy in which the main planks should be co-operation, co-partnership, and self-help. We outlined the departments of the future hospital as including, among others, special ones which had for their main object the supplying of the institution with provisions and apparatus which are now usually procured from outside sources. The ideal of the future—the hospital city—will be a self-dependent organisation, making its own food, its own supplies, and its own apparatus, not merely, in most cases, from raw material gained by purchase from extraneous or at least extra-mural sources, but from raw material provided by itself. By co-operation and co-partnership between two or more of these large institutions which are rapidly coming to be the ideal of the modern hospital, a further economy of energy and material will be effected. The provision of these self-producing departments will render the institutions independent so far as it is possible for them to be so.

Some of our readers have been more than surprised at this forecast. They have jeered at the idea of hospital farms, hospital dairies, hospital poultry yards, hospital abattoirs, hospital carpentering shops, hospital engineering works, hospital ice factories, hospital canning works, and hospital trawlers, just as a former generation laughed at the idea of an institution possessing its own bakery, water plant, electric plant, laundry, or instrument making department. The arguments then advanced against such "extravagant specialisation" hold good to-day as much as they did then. But the arguments in favour of the policy of self-help and co-operation have materially increased. Our object is simply to show our readers what has been done, what is being done, and what is being contemplated. Nor must it be taken for granted that in describing the special lines on which a policy of self-help has progressed at several institutions we are directly approving of such a policy. Obviously a large number of considerations must be taken into account in arriving at a decision, and perhaps the most important consideration is one on which exact information is as yet totally lacking. This is the experience of hospitals which have tried such a policy, at least on progressive lines. No one will deny that it is to the advantage of every large institution to have its own electric plant. Possibly few will deny that it is equally desirable that every large hospital should have its own laundry.

Yet with regard to the latter department it is worth bearing in mind that even the advisability of a separate laundry department for every hospital of large size is not universally admitted. While English, German, and American hospital opinion agrees that a laundry department is an indispensable adjunct to every first-class modern hospital, French, Italian, and Belgian authorities are not so certain on this point. The Brussels hospitals, for example, have co-partnership so far as the laundry is concerned, a central laundry being in existence, which caters for all the city hospitals at a cost which is stated to be much below that of any other large hospital laundry owing to the economy in soap, water, wages, and plant. Similar co-operation has been tried with regard to the milk and meat supply. No hospital, or combination of hospitals, has yet established its own instrument factory, and yet it seems, in view of the fact that the instrument bill of a large hospital amounts to a very appreciable sum annually, that such a step would be justified if the experiment could be made on a co-partnership basis.

Hospital farms have been tried in several instances—we believe in the case of a large London hospital an experiment was made in this direction, but abandoned owing to certain difficulties of an administrative nature. In fact, the whole problem of self-help and co-operation with a view to obtaining independent sources of supply turns on the question of initial expenses. The cost of starting a scheme of supply, even on a co-operative basis, is always considerable. Add to this the opposition by the trade, the competition by outside firms, and the general opinion of the public that hospitals are charitable institutions which need not be run on a business footing, and it is easy to understand the reluctance with which hospital authorities have approached the problem. When they have tackled it, often with most interesting and instructive results, it has generally been owing to the fact that their hands have been forced by an overcharge on the part of contractors or by the delivery of bad material not up to the standard of contract or tender samples.

In the following articles we propose to describe what has been done to secure an independent source of supply in the three main items—namely, meat, milk, and vegetables. We shall also deal briefly with co-operative laundry and pharmacy schemes. The information contained in these articles has been compiled from authentic first-hand sources, and should be of value to all hospital workers because it affords some means of estimating the success or otherwise of the policy which has been adopted by certain institutions. It must be remembered, however, that there are certain very important considerations which cannot accurately be dealt with in a series of brief articles, but which intimately affect the conclusions with regard to the advisability of initiating such a policy. These are local conditions of labour and supply and demand chiefly. With these we cannot deal until we are in a better position to compare the statistics and results obtained abroad with those emanating from the sanatoriums and other institutions in this country.

*(To be continued.)*

